# Downregulation of MCL-1 and upregulation of PUMA using mTOR inhibitors enhance antitumor efficacy of BH3 mimetics in triple-negative breast cancer

**DOI:** 10.1038/s41419-017-0169-2

**Published:** 2018-01-26

**Authors:** Haolong Li, Lei Liu, Haocai Chang, Zhengzhi Zou, Da Xing

**Affiliations:** 1 MOE Key Laboratory of Laser Life Science & Institute of Laser Life Science, College of Biophotonics, South China Normal University, 510631 Guangzhou, China; 2 Joint Laboratory of Laser Oncology with Cancer Center of Sun Yat-sen University, South China Normal University, 510631 Guangzhou, China

## Abstract

Triple-negative breast cancer (TNBC) shows a higher malignant and poorer clinical outcome compared with other breast cancer subtypes. Albeit that chemotherapy is the first choice for TNBC treatment, rapid emergence of chemoresistance and variability of chemotherapeutic responses in TNBC patients call for novel therapeutic strategies. Here, we reported evidences highlighting that combination of BH3 mimetics and mTOR inhibitors could be a promising therapeutic strategy to improve TNBC treatment. Our results showed that combination of the BH3 mimetic ABT263 and typical mTOR inhibitors, BEZ235 or AZD8055, leads to efficient apoptosis in vitro. Tumor regression was significantly improved by combination therapy compared with either drug alone in the xenograft model. Further mechanistic investigations revealed that mTOR inhibitors induced the suppression of MCL-1; concomitantly, the expression level of PUMA was significantly upregulated in a FOXO3a-dependent manner. The specific changes of MCL-1 and PUMA facilitated the release of the apoptotic regulators, such as BIM, BAX, and BAK, to induce the activation of mitochondrial apoptotic pathway, thereby sensitizing the ABT263 activity in TNBC. Therefore, our findings provided evidences that mTOR inhibitors can enhance antitumor efficacy of BH3 mimetics via downregulating MCL-1 and upregulating PUMA in TNBC; it could be a promising therapeutic strategy to treat TNBC.

## Introduction

Triple-negative breast cancer (TNBC), accounting for 15–20% of breast cancers, is defined by lack of expressions of progesterone receptors, estrogen receptors (ERs), and human epidermal growth factor receptor-2 (HER2)^1^. Compared with other breast cancer subtypes, TNBC shows poorer outcome and higher lethality due to an aggressive clinical behavior and a high risk of relapse and metastasis^2^. Although luminal-like breast cancers are sensitive to ER-targeted therapy and HER2-positive breast cancers can benefit from Trastuzumab treatment^3,4^, TNBCs, whose first-line therapy is systematic conventional chemotherapy besides surgery or radiotherapy^5^, has no effective targeted therapy in the clinical setting. Nowadays, certain effect has been observed in TNBC after neoadjuvant chemotherapy^5,6^, but chemotherapy resistance inevitably emerges for the heterogeneity of TNBC, always associated with poor prognosis^1,5^. More importantly, clinical investigations reported that only about 30% of patients suffering TNBC are sensitive to primary chemotherapy and display a pathologic complete response^1,5^, however, patients with residual disease after chemotherapy show high recurrence and a low three-year overall survival rates^6^. Hence, it is urgent to develop new therapeutic strategies that targeting key molecules or signaling pathway members to treat TNBC, which is different from conventional chemotherapy.

In recent years, BCL-2 family proteins, pivotal mediators of apoptosis, especially the pro-survival members, have been focused on as therapeutic targets^7^. BH3 mimetics are inhibitors that could bind to pro-survival members of BCL-2 family and liberate BH3-only proteins that break the balance of anti- vs. pro-survival BCL-2 family proteins to cause apoptosis^7^. It has been reported that directly targeting anti-apoptotic BCL-2 family members by using BH3 mimetics alone or combined with other drugs is an effective therapy for various tumors, such as multiple myeloma, chronic lymphocytic leukemia, colorectal cancer, small-cell lung cancer, and so on^8–12^. For instance, ABT-199, a BCL-2 specific inhibitor approved by FDA, shows great effects in ER-positive breast cancer and different hematological tumors as a single agent^13,14^. Although reports identified that MCL-1 is highly expressed in TNBC following neoadjuvant chemotherapy^5,15^, there is no available approved clinical MCL-1-specific inhibitor that could be used for TNBC treatment. Hence, drugs targeting signaling pathways that regularly control expression of MCL-1 could be considered for treating TNBC. Furthermore, growing evidence has illustrated that simultaneously targeting different pro-survival proteins is a better approach for cancer therapy. Herein, we take it into account that inhibition of MCL-1 in combination with BH3 mimetics may be an effective strategy for TNBC treatment.

AKT/mTOR pathway is a key signaling pathway controlling cell proliferation, protein synthesis, autophagy, and other biological processes^16,17^. Currently, inhibitors targeting key components of AKT/mTOR signaling pathway have been tested in many cancers, even some have applied in preclinical trials^17^. Different studies demonstrated that AKT/mTOR pathway is hyperactive in TNBC^18,19^, so targeting AKT/mTOR pathway using suitable inhibitors is a promising choice for TNBC treatment^1^. Notably, in some cancers, translation of MCL-1 is cap-dependent, which means inhibition of mTOR would suppress MCL-1 by blocking cap-dependent translation. Based on these backgrounds, inhibition of MCL-1 using mTOR inhibitors in combination with BH3 mimetics may show attractive effect in TNBC.

In the present study, we evaluated the combinatorial antitumor effect of BH3 mimetics and mTOR inhibitors in TNBC in vitro and in vivo. We demonstrated that the compound of BEZ235 (mTOR inhibitor) potently enhanced ABT263 (BH3 mimetic)-induced apoptosis of TNBC cell lines, and distinctly repressed tumor growth in TNBC xenografts. Furthermore, we found that BEZ235 suppressing MCL-1 as well as upregulating PUMA contributed to ABT263-induced apoptosis in TNBC. These results provided evidences for the translation of an attractive targeted therapy for TNBC to the clinic.

## Results

### mTOR inhibitors suppress MCL-1 but fail to induce robust apoptosis in TNBC

**Fig. 1:**
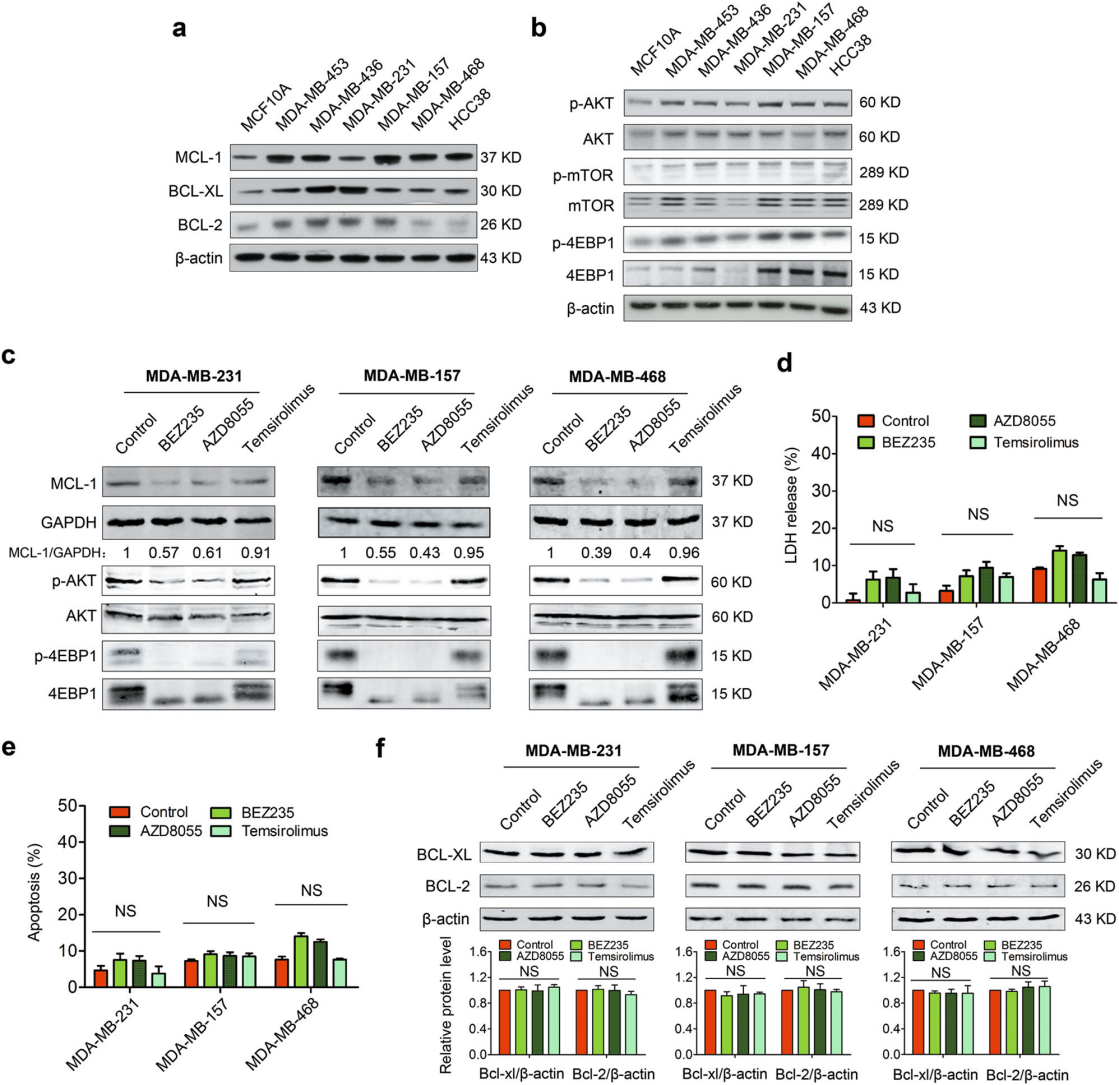
mTOR inhibitors suppress MCL-1 but fail to induce robust apoptosis in TNBC **a** Immunoblotting analysis for basal line expressions of BCL-2 family pro-survival proteins in a panel of TNBC cell lines and MCF10A. β-actin served as a loading control. **b** Immunoblotting analysis for basal line expressions of p-AKT, AKT, p-mTOR, mTOR, p-4EBP1 (NB100-81769), and 4EBP1 (NBP1-47366) in TNBC cells and MCF10A. β-actin served as a loading control. **c** MDA-MB-231, MDA-MB-157, and MDA-MB-468 cells were treated, respectively, with BEZ235 (1 μM), AZD8055 (1 μM), and Temsirolimus (1 μM) for 24 h, then were collected and lysed for immunoblotting analysis using indicated antibodies. **d** LDH release assays of MDA-MB-231, MDA-MB-157, and MDA-MB-468 cells treated with different mTOR inhibitors (1 μM) as indicated for 24 h. Data represents mean ± SEM of three independent experiments. NS means no significant difference vs. control group. **e** Quantity analysis of apoptosis induced by different mTOR inhibitors (1 μM) in MDA-MB-231, MDA-MB-157, and MDA-MB-468 cells. Data represent the mean ± SEM of three independent experiments. NS means no significance. **f** Immunoblotting analysis of expressional changes of BCL-XL and BCL-2 following treating with mTOR inhibitors (1 μM) in MDA-MB-231, MDA-MB-157, and MDA-MB-468 cells. Quantification analysis of the expressions of BCL-XL and BCL-2 is shown in histogram. Data represent the mean ± SEM of three independent experiments. NS means no significant difference.

Overexpression of pro-survival members of BCL-2 family contributes to apoptosis resistance in many cancers^7^. To identify whether pro-survival members were aberrantly expressed in TNBC, we detected protein levels of different members of BCL-2 family in a representative panel of TNBC cell lines. The results showed that the expressions of MCL-1, BCL-XL, and BCL-2 were relatively high in TNBC cell lines (Fig. 1a), especially the MCL-1 level was quite high in most of the tested TNBC cell lines, suggesting that MCL-1 might be a key pro-survival factor in TNBC^15^.

MCL-1 being a key pro-survival factor in TNBC prompted that its inhibition might be an effective method to treat TNBC. Given that currently there is no available approved clinical MCL-1 specific inhibitor, it impelled us to consider other approved drugs targeting key members of signaling pathway controlling MCL-1 expression. Compelling evidence supported that MCL-1 was translated in a cap-dependent manner via mTOR-4EBP1^20,21^, and that AKT/ mTOR pathway was always hyperactive in breast cancer^18,19^. To investigate whether high expression of MCL-1 resulted from hyperactivation of AKT/mTOR pathway in TNBC, immunoblotting was performed. As shown in Fig. 1b, we confirmed that AKT/mTOR cascade proteins (p-AKT, p-mTOR, and p-4EBP1) were highly activated in most of tested TNBC cell lines. Based on this result, we selected three different mTOR inhibitors that have applied in clinical trials^17^—mTORC1/2 inhibitors (BEZ235 and AZD8055) and an mTORC1 inhibitor (Temsirolimus)—to test whether MCL-1 was suppressed when AKT/mTOR pathway was blocked. As expected, we found the mTOR inhibitors, especially the BEZ235 and AZD8055, could significantly inhibit the phosphorylation of AKT and 4EBP1 and efficiently led to decreasing MCL-1 expression (Fig. 1c).

Although mTOR inhibitors could suppress the expression of MCL-1 via blocking cap-dependent translation, whether mTOR inhibitors as single drugs were efficient to kill TNBC cells still needed to be investigated. To this end, LDH release assay was performed to detect the drug cytotoxicity. The results showed that, in different TNBC cells, released LDH after treating with different mTOR inhibitors for 24 h was nearly the same as that in control group (Fig. 1d), indicating that the cytotoxicity of mTOR inhibitors as single agents is limited to treat TNBC cells. It was further confirmed by the result that mTOR inhibitors failed to cause obvious apoptosis in TNBC cells by using annexin V/PI double staining analysis (Fig. 1e and Supplementary Fig. S1).

These results prompted us to raise a question: why were the mTOR inhibitors not sufficient to trigger apoptosis to kill TNBC cells, though mTOR inhibitors could suppress MCL-1 expression, a pivotal anti-apoptotic protein in TNBC? To answer this question, immunoblotting was performed to test the expressions of other important BCL-2 pro-survival members following treatments with mTOR inhibitors. Despite that MCL-1 was suppressed by mTORC1/2 inhibitors, no obvious changes of BCL-2 and BCL-XL expression were detected (Fig. 1f), implying that BCL-2 or BCL-XL would be attributed to apoptosis resistance once MCL-1 was inhibited. Therefore, these results prompted us to consider BH3 mimetics, specially designed to target anti-apoptotic proteins, like ABT263, in combination with mTOR inhibitors might trigger apoptosis to treat TNBC.

### Suppressing MCL-1 sensitizes TNBC cells to ABT-263

**Fig. 2:**
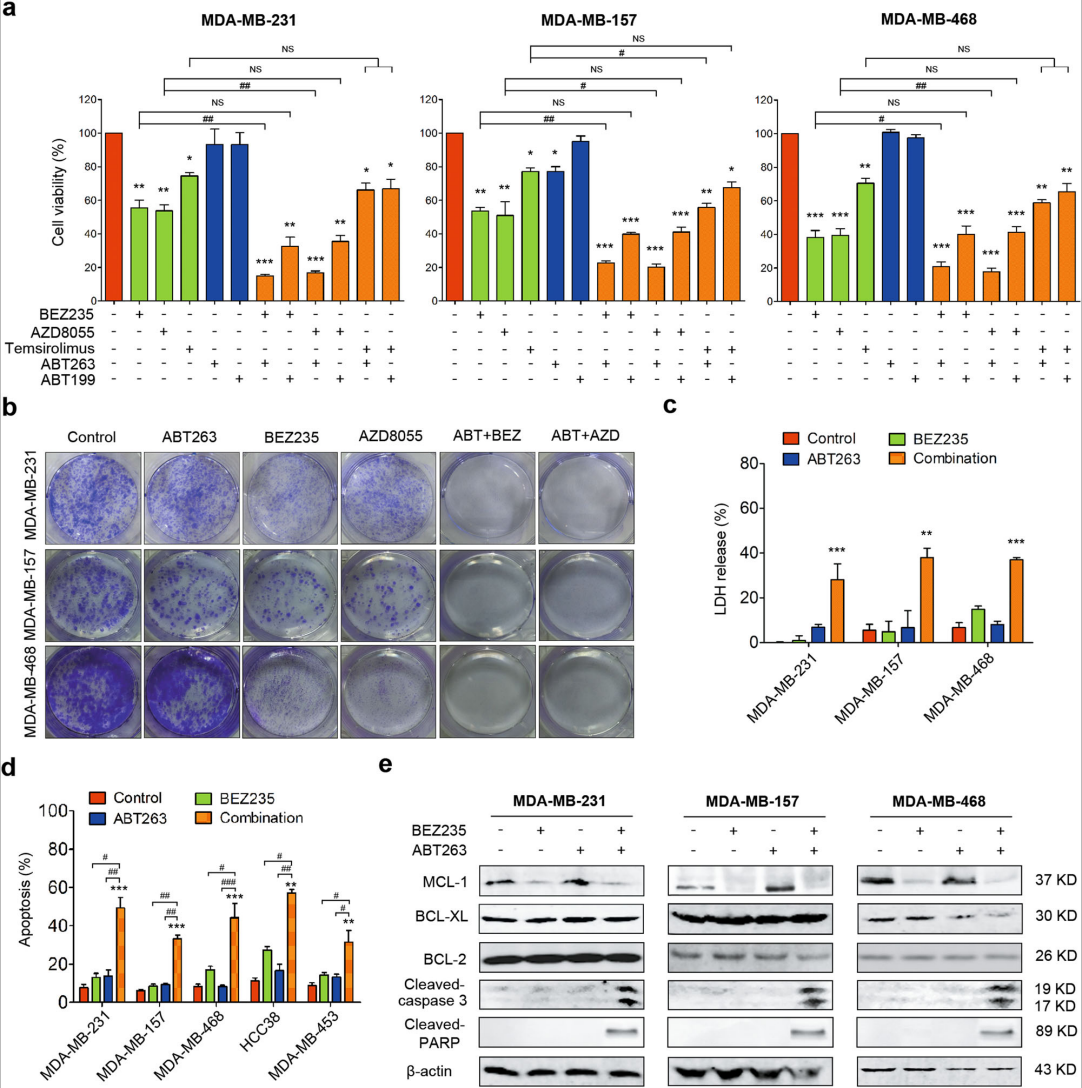
Suppression of MCL-1 sensitizes TNBC cells to ABT-263 **a** Cell viability analysis of MDA-MB-231, MDA-MB-157, and MDA-MB-468 cells treated with indicated drug combinations for 24 h using CCK-8 kit. mTOR inhibitors (BEZ235, AZD8055, and Temsirolimus) and BH3 mimetics (ABT263 and ABT199) were supplied at 1 μM. Data represent the mean ± SEM of three independent experiments. ^*^P *<* 0.05, ^**^P *<* 0.01 and ^***^P *<* 0.001 vs. control group; ^#^*P* < 0.05 and ^##^*P* < 0.01 vs. indicated group; NS means no significance vs. indicated group. **b** Representative images of colony formation assays of MDA-MB-231, MDA-MB-157, and MDA-MB-468 cells treated with 1 μM BEZ235 or AZD8055 in combination with ABT263 (1 μM). **c** LDH release assays of MDA-MB-231, MDA-MB-157, and MDA-MB-468 cells following exposure to no drug (control), BEZ235 (1 μM), and/or ABT263 (1 μM) for 24 h. Data represent the mean ± SEM of three independent replicates. ^**^P *<* 0.01 and ^***^P *<* 0.001 vs. indicated control group. **d** Quantity analysis of apoptotic tests in different TNBC cells treated with no drug (control), BEZ235 (1 μM), and/or ABT263 (1 μM) for 24 h. Data represent the mean ± SEM of three independent experiments. ^**^P *<* 0.01 and ^***^P *<* 0.001 vs. indicated control group; ^#^*P* < 0.05, ^##^*P* *<* 0.01 and ^###^*P* *<* 0.001 vs. indicated group. **e** Immunoblotting analysis of expressional changes of MCL-1, BCL-XL, and BCL-2 as well as cleaved-caspase 3 and cleaved-PARP following treatments with no drugs (control), BEZ235 (1 μM) and/or ABT263 (1 μM) in MDA-MB-231, MDA-MB-157, and MDA-MB-468 cells.

Based on the obtained data, we hypothesized that mTOR inhibitors in combination with BH3 mimetics could be efficient to treat TNBC. Hence, we evaluated the effect of combination therapy of mTOR inhibitors and BH3 mimetics in TNBC. Firstly, the cell viability following indicated treatments was observed and the result showed that mTORC1/2 inhibitors (BEZ235 or AZD8055) in combination with ABT263 significantly inhibited cell viability in MDA-MB-231, MDA-MB-157, and MDA-MB-468 cells, comparing with each compound treatment alone (Fig. 2a). But treatment with Temsirolimus in combination with ABT263 or ABT199 only induced modest cell viability inhibition (Fig. 2a). To directly observe whether combination therapy shows a long-term effect in TNBC, colony formation assays were performed. The results showed that AZD8055 or BEZ235 in combination with ABT263 strongly inhibited cell proliferation in different TNBC cell lines (Fig. 2b). In order to confirm whether there is a synergistic effect of ABT263 and mTORC1/2 inhibitors (BEZ235 or AZD8055), combination index (CI) values were calculated using the Chou–Talalay method^22^. Our results showed that ABT263 had an evident synergistic effect with mTORC1/2 inhibitors in tested TNBC cells (Table 1).

**Table 1:** ABT263 in combination with BEZ235 or AZD8055 shows a synergistic efficacy in TNBC

Cell line	ABT263 (μM)	BEZ235 (μM)	CI	*R* value	ABT263 (μM)	AZD8055 (μM)	CI	*R* value
MDA-MB-231	0.25	0.25	0.278		0.25	0.25	0.131	
	0.50	0.50	0.3504		0.50	0.50	0.1448	
	1.00	1.00	0.2429	0.94	1.00	1.00	0.0492	0.98
	2.00	2.00	0.2661		2.00	2.00	0.0616	
	4.00	4.00	0.0361		4.00	4.00	0.0209	
MDA-MB-157	0.25	0.25	0.1406		0.25	0.25	0.3203	
	0.50	0.50	0.2401		0.50	0.50	0.1838	
	1.00	1.00	0.3437	0.98	1.00	1.00	0.274	0.97
	2.00	2.00	0.4463		2.00	2.00	0.486	
	4.00	4.00	0.7333		4.00	4.00	0.7998	
MDA-MB-468	0.25	0.25	0.0748		0.25	0.25	0.0877	
	0.50	0.50	0.0466		0.50	0.50	0.0376	
	1.00	1.00	0.0604	0.84	1.00	1.00	0.0685	0.87
	2.00	2.00	0.1089		2.00	2.00	0.1291	
	4.00	4.00	0.2451		4.00	4.00	0.2861	

Combination index (CI) values were calculated using Compusyn software. CI < 1 means synergism, CI = 1 respresents additive effects, and CI > 1 indicates antagonism

Next, we performed LDH release assays to determine the cytotoxicity of this combination therapy; the results suggested that the combination therapy could remarkably kill TNBC cells (Fig. 2c). Additionally, apoptotic analysis showed combination therapy of ABT263 and BEZ235 gave rise to much more cell death than mono-treatments (Fig. 2d and Supplementary Fig. S2). Furthermore, in different TNBC cells, obvious expressions of cleaved-caspase 3 and cleaved-PARP, vital apoptotic markers, were also detected in the context of combination therapy, comparing to mono-treatments (Fig. 2e). In total, these data suggest combinational therapy of mTORC1/2 inhibitors and ABT263 is a potential therapeutic strategy for TNBC.

### BEZ235 upregulates PUMA in a FOXO3a-dependent manner and downregulates MCL-1 at translational level

**Fig. 3:**
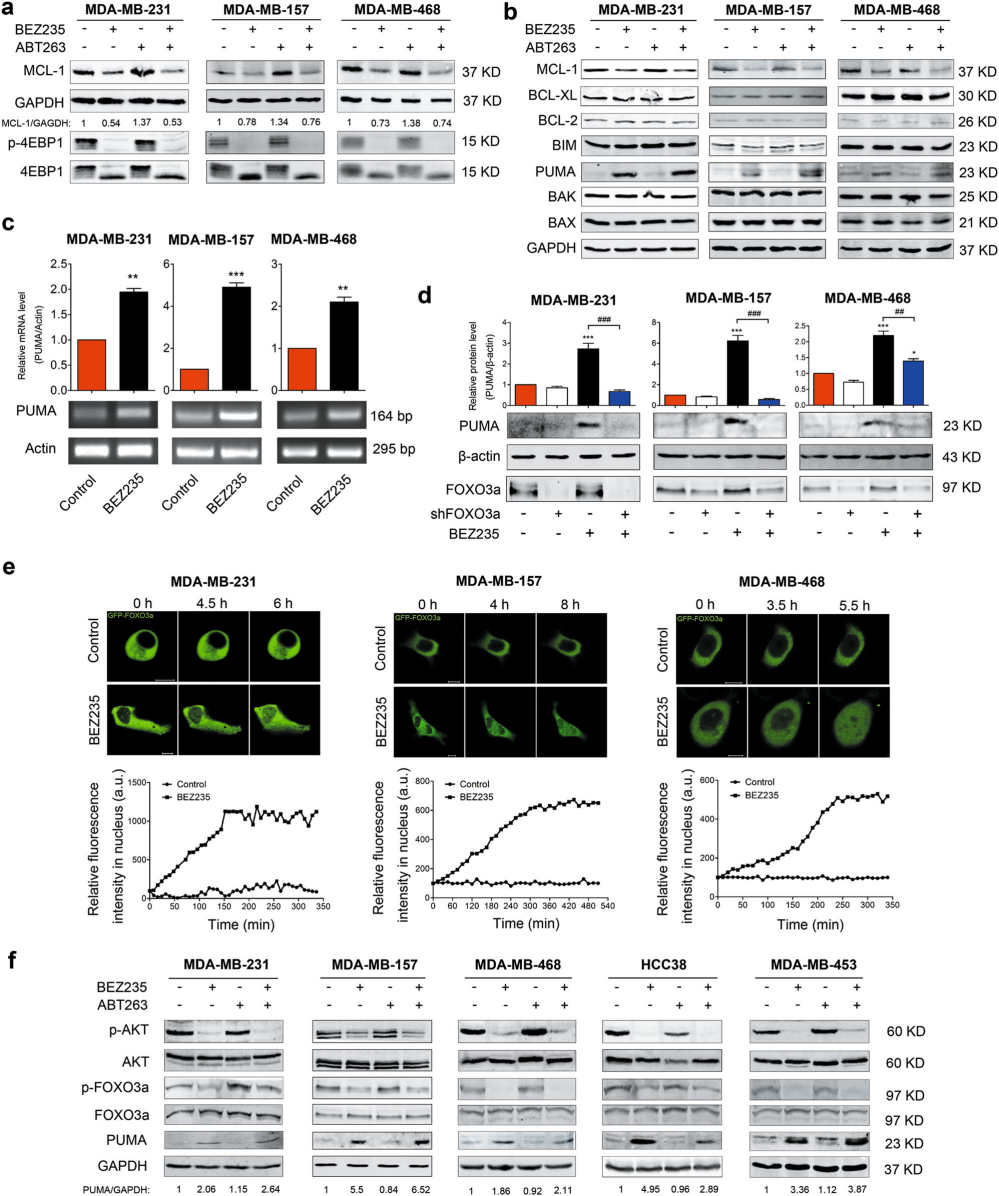
BEZ235 downregulates MCL-1 at translational level, whereas upregulates PUMA in FOXO3a-dependent manner **a** Immunoblotting analysis of MCL-1 and p-4EBP1 levels in MDA-MB-231, MDA-MB-157, and MDA-MB-468 cells following exposure to no drug (control), BEZ235 (1 μM), and/or ABT263 (1 μM) for 24 h. **b** Immunoblotting analysis of expressional changes of different members of BCL-2 family in MDA-MB-231, MDA-MB-157, and MDA-MB-468 cells after treating with no drug (control), BEZ235 (1 μM) and/or ABT263 (1 μM) for 24 h. **c** Analysis of *PUMA* mRNA levels following BEZ235 treatment in MDA-MB-231, MDA-MB-157, and MDA-MB-468 cells. Quantification analysis is shown in the histogram. Data represent the mean ± SEM of three independent experiments. ^**^P < 0.01 and ^***^P < 0.001 vs. control group. **d** Knockdown of FOXO3a blocked PUMA increase induced by BEZ235. After transfection of FOXO3a shRNA for 48 h, MDA-MB-231, MDA-MB-157, and MDA-MB-468 cells were, respectively, treated with or without BEZ235 (1 μM) for 24 h, and then subjected to immunoblotting analysis. Quantification analysis is shown in the histogram. Data represent the mean ± SEM of three independent experiments. ^*^P < 0.05 and ^***^P < 0.001 vs. control group; ^##^*P* < 0.01, ^###^*P* < 0.001 vs. indicated group. **e** BEZ235 induced GFP-FOXO3a translocation from cytoplasm to nucleus. MDA-MB-231, MDA-MB-157, and MDA-MB-468 cells were transfected with GFP-FOXO3a plasmid for 48 h and then dynamically monitored its temporal and spatial changes by treating with BEZ235. **f** Immunoblotting analysis of expressional changes of p-AKT, p-FOXO3a, and PUMA in different TNBC cell lines following treating with no drug (control), BEZ235 (1 μM), and/or ABT263 (1 μM) for 24 h.

These findings motivated us to further explore the molecular mechanisms of the combination therapy of BEZ235 and ABT263 in TNBC. As described above, mTORC1/2 inhibitors inhibited the expression of MCL-1 in MDA-MB-231 cells (Fig. 1c). We further tested whether the compound BEZ235 had the same effect on other TNBC cell lines. Consistent with the results in Fig. 1c, in different TNBC cell lines, MCL-1 was also downregulated by BEZ235 (Fig. 3a refers to MDA-MB-231, MDA-MB-157, and MDA-MB-468, and Supplementary Fig. S3a refers to HCC38 and MDA-MB-453). Concomitantly, significant inhibitions of p-4EBP1 and p-AKT were also detected (Fig. 3a, f and Supplementary Fig. S3a), suggesting that protein translation was blocked to suppress MCL-1 expression.

To further reveal the mechanism, immunoblotting was performed to investigate whether the expressions of other BCL-2 family members were altered in these experimental conditions. Of note, we found that, besides MCL-1, the expression of PUMA but not other members was significantly increased following treatment of BEZ235 with or without ABT263 in MDA-MB-231, MDA-MB-157, and MDA-MB-468 cells (Fig. 3b). We then detected PUMA expression with exposure to multiple mTOR inhibitors in MDA-MB-231 and the result further confirmed that PUMA could be upregulated by mTOR inhibitors (Supplementary Fig. S3b).

Then we investigated the reason why PUMA was increased following the treatment of mTOR inhibitors. PUMA as an important pro-apoptotic member involving in the treatment of many cancers^23–25^, which can be induced in p53-dependent and p53-independent manners under various stimuli^26,27^. Notably, FOXO3a is a transcription factor controlling transcription of many genes including *PUMA*^27^. It prompted us to consider that FOXO3a might be an important regulator involved in induction of PUMA following exposure to BEZ235. Our results showed that the transcriptional expression of *PUMA* was significantly increased in different TNBC cells treated by BEZ235 (Fig. 3c); while knockdown of FOXO3a significantly blocked the increase of PUMA (Fig. 3d). Further study showed that BEZ235 promoted the translocation of GFP-FOXO3a from cytoplasm to nucleus to play its transcriptional activity (Fig. 3e). As expected, BEZ235 blocked the activation of AKT to dephosphorylate FOXO3a, enhancing its activity; while PUMA was obviously increased concomitantly in other detected TNBC cells (Fig. 3f). These findings suggested that BEZ235 promoted PUMA expression in a FOXO3a-dependent manner.

### Downregulation of MCL-1 and upregulation of PUMA induced by BEZ235 contributes to ABT263-induced apoptosis

**Fig. 4:**
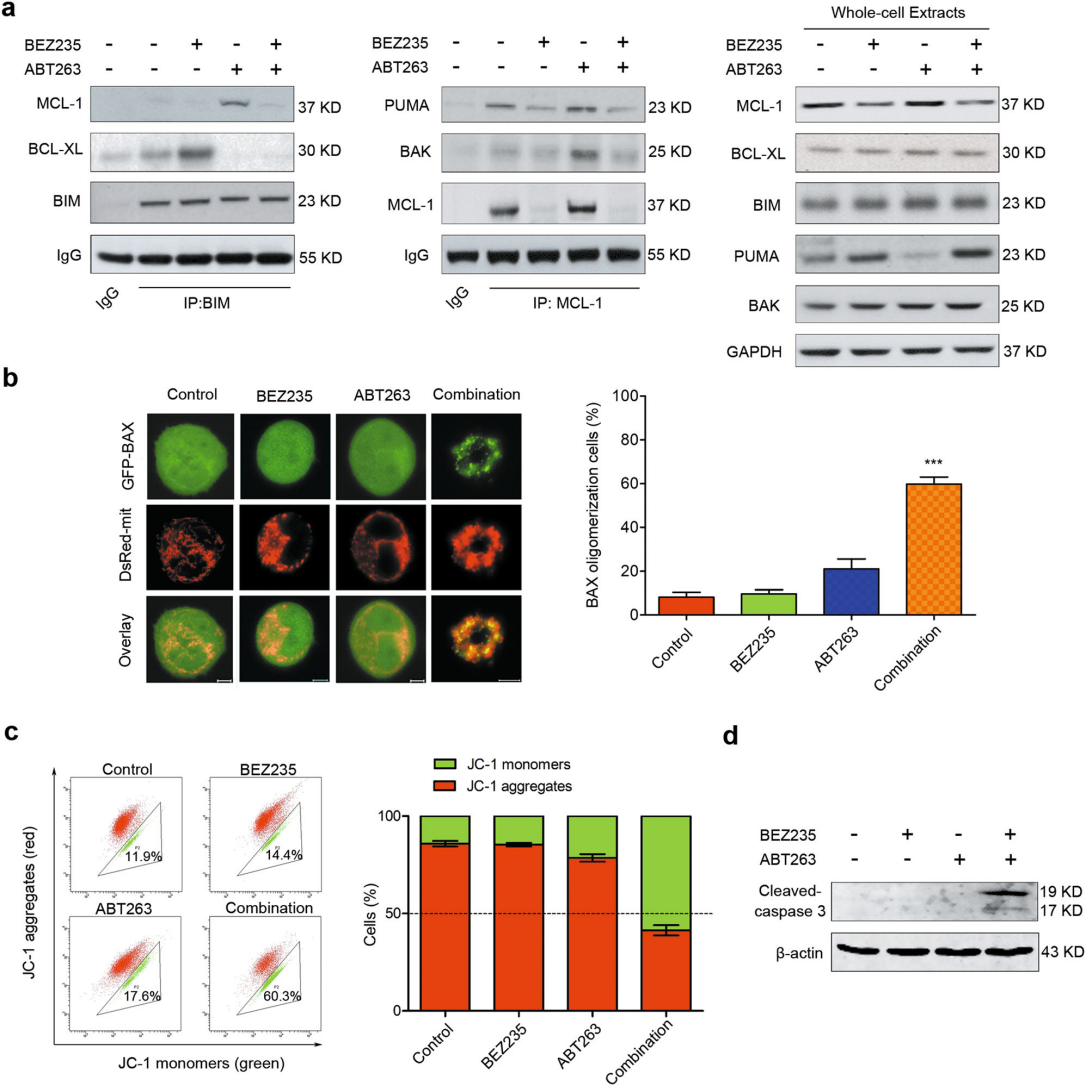
Downregulation of MCL-1 and upregulation of PUMA induced by BEZ235 contributes to ABT263-induced apoptosis **a** CO-IP analysis of interactions between BIM and BCL-XL or MCL-1 (left); and interactions between MCL-1 and PUMA or BAK in MDA-MB-231 following treatments with no drug, BEZ235 (1 μM), and/or ABT263 (1 μM) for 24 h (middle). IgG in first lane as a negative control. Whole-cell extracts were analyzed using indicated antibodies (right). **b** BAX translocation was observed using confocal microscopy in MDA-MB-231 cells after treated with no drug, BEZ235 (1 μM), and/or ABT263 (1 μM) for 24 h. Typical images are displayed (left), bar = 5 μm. Quantification of cells showing BAX oligomerization (right). After different treatments as indicated, the percentage of cells showing BAX oligomerization was assessed by counting the number of cells exhibiting mitochondrial BAX. Data represent the mean ± SEM of three independent experiments. ^***^P < 0.001 vs. control group. **c** Mitochondrial membrane potential was determined using JC-1 staining in MDA-MB-231 cells following treatments with no drug, BEZ235 (1 μM), and/or ABT263 (1 μM) for 24 h. Quantification analysis of cells showing changes in mitochondrial membrane potential (JC-1 red to green) is shown in the histogram (right). **d** Immunoblotting analysis of expressional changes of cleaved-caspase 3 in MDA-MB-231 cells following treating with no drug (control), BEZ235 (1 μM), and/or ABT263 (1 μM) for 24 h.

We then performed co-immunoprecipitation assays to investigate the dynamic changes of the protein complexes of BCL-2 family members during apoptosis induced by combination therapy in TNBC. Previous studies illustrated that ABT263 could induce apoptosis in which BIM is involved as an important apoptotic regulator^28,29^. We found that single treatment with ABT-263 disrupted the complexes of BCL-XL and BIM, while the amount of MCL-1 binding to BIM increased markedly (Fig. 4a, left). Additionally, the interaction between MCL-1 and BAK was also significantly increased following mono-treatment of ABT263 (Fig. 4a, middle). However, BIM and BAK were obviously released to trigger apoptosis once MCL-1 was suppressed by adding BEZ235 in the presence of ABT-263. Of note, although PUMA was upregulated by BEZ235 (Fig. 4a, right), less PUMA was bound to MCL-1 than control group due to inhibition of MCL-1 (Fig. 4a, middle). Thus redundant PUMA could promote apoptosis following the combination therapy.

Activation of BAX or BAK led to mitochondrial outer membrane permeabilization (MOMP) is a critical event during apoptosis^7,30^. To observe redistribution of BAX during apoptosis induced by combination treatment of BEZ235 and ABT263, GFP-BAX and DsRed-mit plasmids were transiently transfected into MDA-MB-231 cells. GFP-BAX was used to observe BAX migration, and DsRed-mit was used to label mitochondria. Overlay images displayed in Fig. 4b showed that, compared to mono-treatments, combination treatment obviously caused BAX translocation to the mitochondria, indicating that BAX was activated. The temporal and spatial changes in BAX subcellular localization were also assessed in a real-time manner (Supplementary Fig. S4a, b). The results showed that obvious translocation of BAX was observed at about 6 h following combination treatment, however, it occurred after 20 h with ABT263 mono-treatment, suggesting that combination treatment was more efficient to induce apoptosis than mono-treatment with either drug (Supplementary Fig. S4a, b). To directly observe MOMP during apoptosis, changes of mitochondrial membrane potential were detected using JC-1 staining. The results showed that combination treatment caused sharp loss of mitochondrial membrane potential, compared with mono-treatments (Fig. 4c). Finally, caspase 3 was activated as evidenced by cleaved-caspase 3 expression in combination treatment group (Fig. 4d).

### The combination therapy of BEZ235 and ABT263 potently represses tumor growth in vivo

**Fig. 5:**
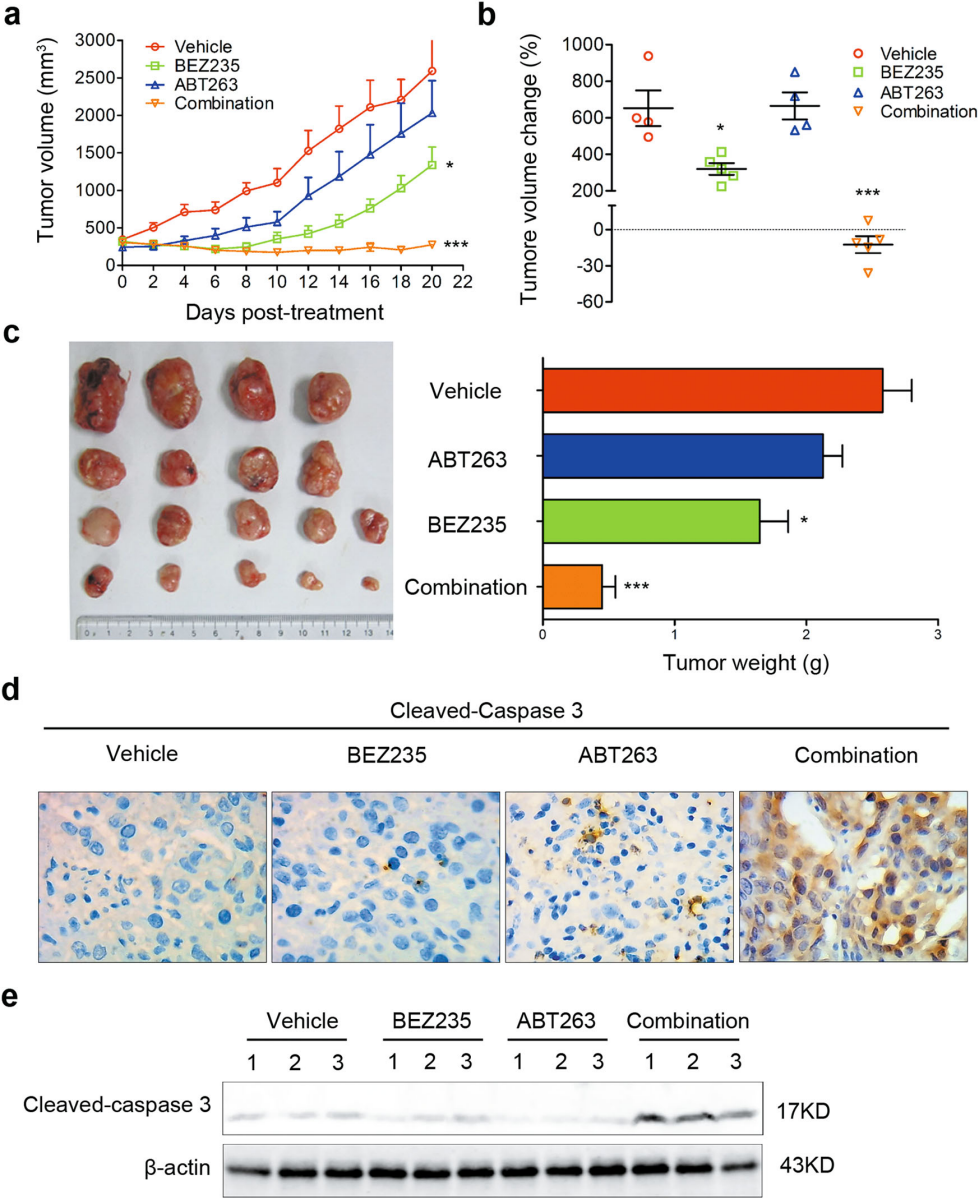
The combination therapy of BEZ235 and ABT263 potently represses tumor growth in vivo **a** Tumor-bearing nude mice were treated with vehicle, BEZ235 (15 mg/kg) and/or ABT263 (25 mg/kg) for 4 weeks, and tumor volumes were measured every 2 days over 3 weeks. Error bar, mean ± SEM (*n* ≥ 4 per group). ^*^P < 0.05 and ^***^P < 0.001 vs. vehicle group. **b** Analysis of tumor volume changes in mice after different treatments as in (A). Error bar, mean ± SEM (*n* ≥ 4 per group). ^*^P < 0.05 and ^***^P < 0.001 vs. vehicle group. **c** Representative images of tumors collected from tumor-bearing nude mice following treatments as indicated for 4 weeks (left). Quantification analysis of tumor weights is shown in histogram (right). Error bar, mean ± SEM (*n* ≥ 4 per group). ^*^P < 0.05 and ^***^P *<* 0.001 vs. vehicle group. **d** Immunohistochemical (IHC) analysis for changes of cleaved-caspase 3 expression in harvested tumors following different treatments as indicated. Representative images are shown (magnification: ×40). **e** Immunoblotting analysis of expressional changes of cleaved-caspase 3 in collected tumors from different groups. Each lane in indicated group represents a tumor sample collected from an individual mouse.

To further evaluate the effect of combination therapy of BEZ235 and ABT263 in TNBC in vivo, a TNBC xenograft tumor model was established using MDA-MB-231 cell line. In this model, BEZ235 was administrated i.g. at a daily dose of 15 mg/kg and ABT263 was injected i.p. at a dose of 25 mg/kg three times per week. Our results showed that combination therapy of BEZ235 and ABT263 significantly suppressed tumor growth, comparing with each drug treatment alone (Fig. 5a–c). Further study showed that combination treatment induced obvious expression of cleaved-caspase 3, an essential apoptotic effector (Fig. 5d, e and Supplementary Fig. S5a). These results demonstrated that combination therapy of BEZ235 and ABT263 was an effective method for TNBC treatment in vivo.

We also tested toxic side effects of combination therapy in this model. Result of monitoring body weights showed that there was no significant difference between vehicle and combination therapy group (Supplementary Fig. S5b); and hematoxylin–eosin staining results also showed no tissue alterations in heart, lung, kidney, liver, and spleen following combination treatment (Supplementary Fig. S5c), indicating that organ-toxic effects of this combination therapy are limited.

**Fig. 6:**
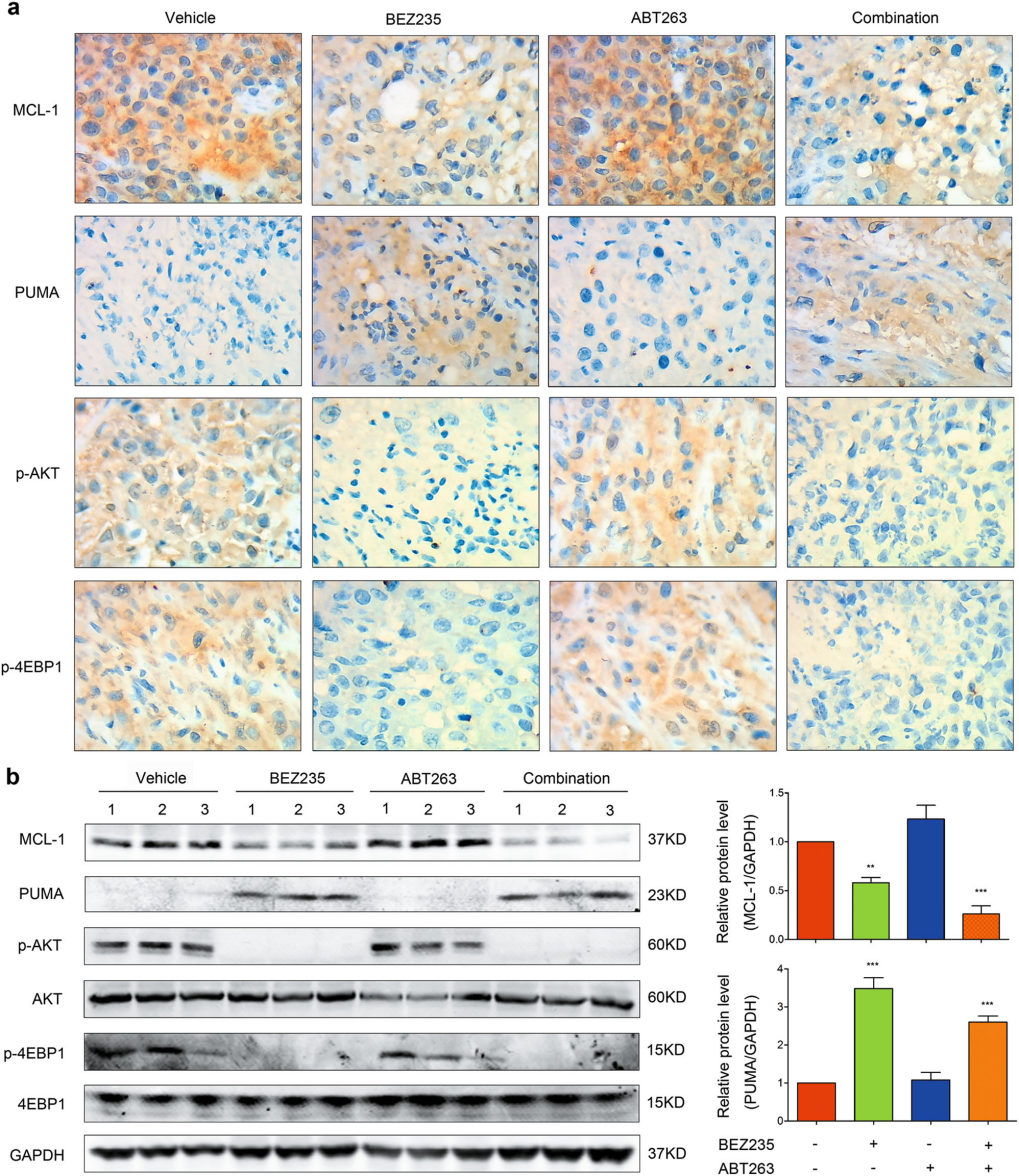
Combination of BEZ235 and ABT263 alters the expressions of MCL-1 and PUMA in vivo **a** IHC analysis of expressional changes of MCL-1, PUMA, p-AKT, and p-4EBP1 in tumors collected from nude mice following treatments with vehicle, BEZ235 (15 mg/kg) and/or ABT263 (25 mg/kg) for 4 weeks. Representative images are shown (magnification: ×40). **b** Immunoblotting analysis of the expressions of MCL-1, PUMA, p-AKT, and p-4EBP1 in tumors from different groups (left). Each lane in indicated group represents a tumor sample collected from an individual mouse. Quantification analyses of the expressions of MCL-1 and PUMA are shown in histogram (right). Error bar, mean ± SEM (*n* = 3). ^**^P < 0.01, ^***^P < 0.001 vs. vehicle group.

Then we tested whether the expressions of MCL-1 and PUMA were altered in vivo, as in vitro. The results showed that, following BEZ235 treatment with or without ABT263, the expression of MCL-1 was suppressed while PUMA was increased; correspondingly, the expressions of p-AKT and p-4EBP1 were significantly suppressed (Fig. 6a, b). Thus, these results demonstrated that combination of BEZ235 and ABT263 is an effective and safe therapy for TNBC in vivo.

## Discussion

**Fig. 7:**
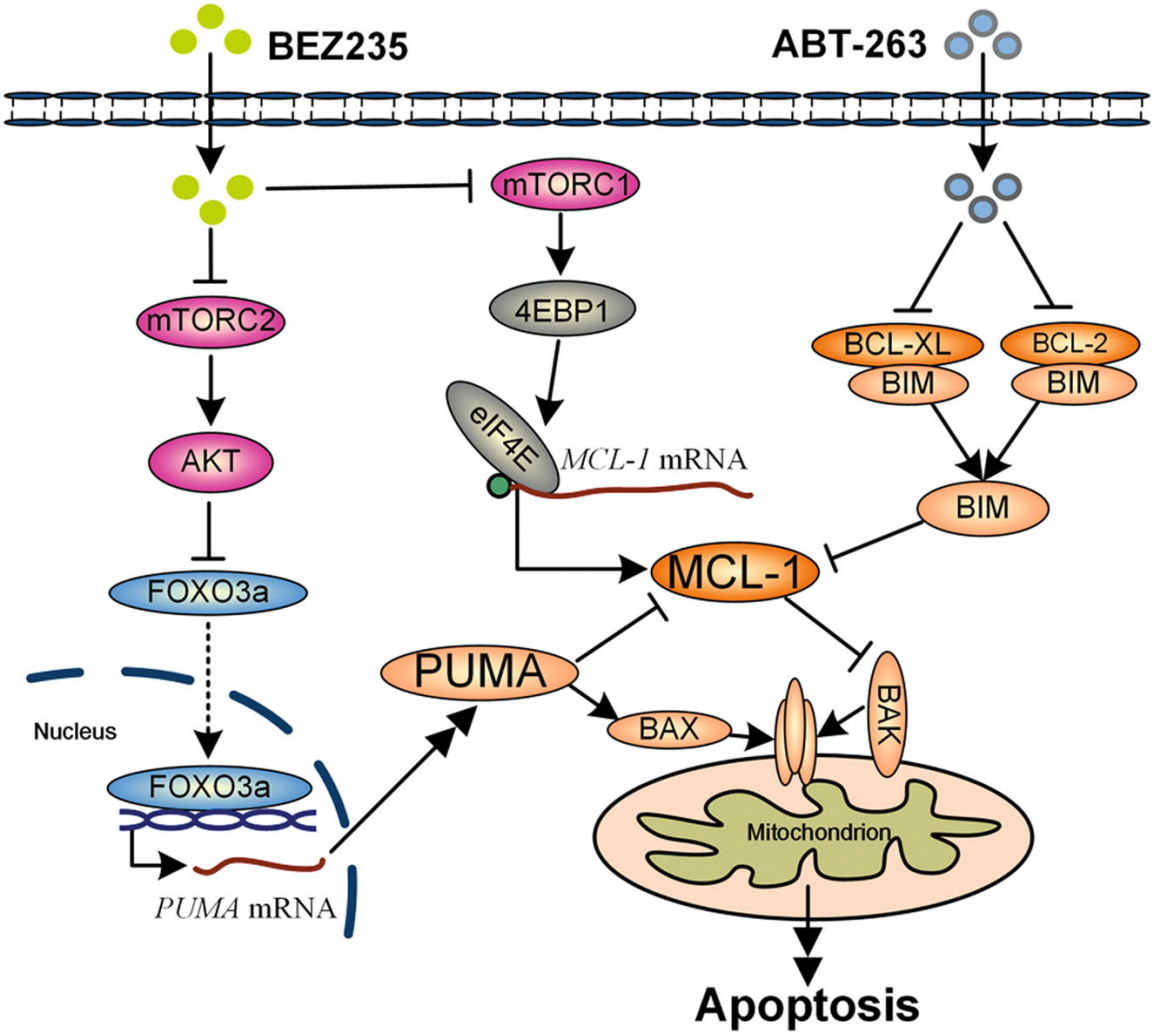
Schematic representation of molecular mechanism of combination therapy of BEZ235 and ABT263 mTOR inhibitor (BEZ235) suppresses mTORC1 activity, thereby inhibiting 4EBP1/eIF4E-regulated translation of MCL-1; simultaneously, inhibition of mTORC2 by BEZ235 decreases the phosphorylation of AKT, promoting FOXO3a nuclear translocation by suppressing its phosphorylation, leading to upregulation of PUMA. ABT263, a BH3 mimetic targeting BCL-2 and BCL-XL, but not MCL-1, can disrupt the complexes of BIM/BCL-XL and BIM/BCL-2, releasing BIM to bind MCL-1. Therefore, the combination therapy of BEZ235 and ABT263 breaks the balance of BCL-2 family members, triggering effective apoptosis.

TNBC is the most intractable subtype in breast cancer, always shows poor prognosis and high risk of recurrence clinically^1^. Due to lack of effective therapeutic targets, conventional chemotherapy is the main treatment for TNBC in most instances^1,2^. Recently, many efforts were made based on chemotherapy, like neoadjuvant chemotherapy, and some benefits were shown in TNBC;^5,31^ however, chemotherapy resistance invariably develops due to its heterogeneity^5^. Given that 60–70% of patients suffering TNBC are not fully sensitive to chemotherapy^1,5^, new therapeutic strategies targeting key survival proteins or regulators needed to be developed for TNBC treatment. Indeed, many agents targeting key molecules in TNBC have been studied, such as MEK inhibitors, PARP inhibitors, PI3K inhibitors, and HSP90 inhibitors^32–34^. Despite many efforts, more novel therapies or combination paradigms still needed to be developed, which is based on the reason that which method is more effective and safe for TNBC that has not been evaluated clinically. Hence, in the present study, combination of mTOR inhibitors and BH3 mimetics as a potential therapeutic paradigm was studied for TNBC treatment. We found that combination of BEZ235 and ABT263 could significantly induce apoptosis in vitro and in vivo, of which the mechanism is mTOR inhibitors suppressing MCL-1 and upregulating PUMA promotes ABT263 to activate mitochondrial apoptotic pathway in TNBC (Fig. 7).

MCL-1 has been reported as a pivotal pro-survival member in various cancers^12,15^. Consistent with this notion, we found MCL-1 was highly expressed in TNBC cell lines (Fig. 1a). Although we showed for the first time that mTORC1/2 inhibitors downregulated MCL-1 in TNBC cells, mTOR inhibitors as single agents were not cytotoxic enough, and consequently failed to induce obvious apoptosis (Fig. 1c–e), which is possibly due to high BCL-XL level is resistant to MCL-1 inhibition^35^. Previous investigations revealed that inhibitions of MCL-1 and BCL-XL/BCL-2 were effective in cancers^8,36^. The data in current study demonstrated that suppressing MCL-1 using mTORC1/2 inhibitors in combination with BCL-XL/BCL-2 inhibitors showed promising effects in TNBC cells (Fig. 2 and Supplementary Fig. S2). In this study, only the approved mTORC1/2 inhibitors were selected to suppress MCL-1. Nevertheless, we believed that MCL-1 specific inhibitors, like S63845 which is recently reported as an MCL-1 specific inhibitor and displayed great effect in diverse cancers^37^, will also be effective as a single drug or in combination with ABT263 in TNBC, notwithstanding further investigations will be needed.

In the present study, we revealed that AKT/mTOR pathway was hyperactive in TNBC cells (Fig. 1b). Given that AKT/mTOR pathway controlled various biological processes, including protein synthesis and cell proliferation^17^, our combinatorial therapeutic model of mTORC1/2 inhibitors and BH3 mimetics could also induce cell growth arrest besides apoptosis (Figs. 2 and 5), which will be more attractive than the therapies only aimed at induction of apoptosis. In the current study, although mTORC1/2 inhibitors in combination with BCL-2/BCL-XL inhibitors showed exciting effect for TNBC treatment, mTORC1 inhibitor in combination with ABT263 was not effective enough (Fig. 2a). We supposed that the reason why mTORC1 inhibitor in combination with ABT263 could not induce substantial inhibition of cell viability is possibly that mTORC1 inhibitor failed to decrease expression of MCL-1 due to activation of AKT by mTORC2 despite inhibition of mTORC1, consistent with previous reports^17^.

It has been reported that, besides MCL-1, mTORC1/2 inhibitors could alter the expressions of other BCL-2 family members in different cancer cell lines^24,38^. Although investigations reported that BIM was upregulated in some cancers^36,38^, we failed to detect the increase of BIM following combination therapy of BEZ235 and ABT263 in TNBC. In our current study, the data showed that mTORC1/2 inhibitors induced PUMA in a FOXO3a-dependent manner in TNBC cells (Fig. 3b–f and Supplementary Fig. S3b). As expected, co-immunoprecipitation analysis confirmed that PUMA played an important role in apoptosis induced by combination therapy (Fig. 4a).

Overall, our study demonstrated that MCL-1 was a key therapeutic target of TNBC, and offered a promising combination paradigm of targeting MCL-1 and BCL-XL/BCL-2 for TNBC treatment. To facilitate drug development and clinical translation, mechanistic evidence to this therapeutic strategy was also expounded. mTOR inhibitor BEZ235 could enhance the antitumor efficacy of ABT263 by upregulating PUMA and downregulating MCL-1 to affect the interaction balance of BCL-2 family members. Therefore, the BEZ235 and ABT263 combination therapy was more effective than each drug treatment alone (Fig. 7). Based on the fact that the combination therapeutic strategy showed great effect in TNBC, we inferred that it would also be effective in those cancers showing the same molecular profiles as TNBC, especially high expression of MCL-1 controlled by AKT/mTOR pathway. Our findings provided evidences that the combination of BH3 mimetics with mTOR inhibitors can lead to efficient apoptosis in TNBC; it could be a promising therapeutic strategy to treat these refractory cancers in the future.

## Materials and methods

### Cell lines

All cell lines except MCF10A were obtained from the Cell Bank of Type Culture Collection of the Chinese Academy of Sciences (Shanghai, China). MCF10A was from Sun Yet-sen University as a generous gift. All cell lines used were authenticated by short-tandem repeat profiling. The cell lines of MDA-MB-157, MDA-MB-453, and MDA-MB-468 were cultured in Leibovitz’s L-15 Medium with 10% fetal bovine serum (FBS, Gibco, Australia); MDA-MB-436 was cultured in Leibovitz’s L-15 medium supplemented with 10% FBS, 10 μg/ml insulin (Sigma-Aldrich), and 16 μg/ml glutathione (Sigma-Aldrich); MDA-MB-231 was cultured in Dulbecco modified Eagle medium (DMEM) with 10% FBS, and HCC38 was grown in PRMI Medium with 10% FBS. MCF-10A cells were cultured in DMEM/F12 (1:1) medium with 5% horse serum (Hyclone), 10 μg/ml insulin (Sigma-Aldrich), 20 ng/ml epidermal growth factor (EGF; Peprotech), 0.5 μg/ml hydrocortisone (Sigma-Aldrich), and 100 ng/ml cholera toxin (Sigma-Aldrich). All cell lines mentioned above were grown in the presence of penicillin (100 U/ml) and streptomycin (0.1 mg/ml).

### Antibodies and chemicals

Antibodies used for immunoblotting analyses in this study are listed below: rabbit anti-MCL-1 (1:500, ab32087), rabbit anti-BAX (1:1000, ab32503) were obtained from Abcam; mouse anti-BCL-2 (1:1000, #15071), rabbit anti-BIM (1:1000, #2933), rabbit anti-BCL-XL (1:1000, #2762), rabbit anti-BAK (1:1000, #12105), rabbit anti-AKT (1:1000, #4691), rabbit anti-p-AKT (1:1000, #4060), rabbit anti-mTOR (1:1000, #2983), rabbit anti-p-mTOR (1:1000, #5536), rabbit anti-p-4EBP1 (1:1000, #2855), rabbit anti-4EBP1 (1:1500, #9644), rabbit anti-FOXO3a (1:1000, #12829), rabbit anti-p-FOXO3a (1:1000, #9466) rabbit anti-cleaved PARP (1:1000, #5625) and rabbit anti-cleaved Caspase-3 (1:1000, #9664) were purchased from Cell Signaling Technology; mouse anti-β-actin (1:1000, sc-47778), mouse anti-GAPDH (1:1000, sc-32233) and rabbit anti-PUMA (1:1000, sc-28226) were from Santa Cruz Biotechnology; rabbit anti-p-4EBP1 (1:500, NB100-81769, used in Fig. 1b) and mouse anti-4EBP1 (1:1000, NBP1-47366, used in Fig. 1b) were obtained from Novus Biologicals. The compounds of BH3 mimetics (ABT263 and ABT199) and mTOR inhibitors (BEZ235, AZD8055, and Temsirolimus) were from AbMole Bioscience.

### Immunoblotting and co-immunoprecipitation

Immunoblotting was performed as previously described^39^. Briefly, cells after treatments were collected and lysed using NP40 lysis buffer (P0013F; Beyotime Biotechnology) supplemented with inhibitor cocktail (cat. no. 05892791001, Roche) for 60 min on ice. Equivalent proteins were loaded on SDS-PAGE and transferred to PVDF membranes, then incubated with primary antibodies at 4 °C overnight or at room temperature for 2 h after blocking with 5% non-fat milk, followed by indicated Alexa Fluor-conjugated secondary antibodies for 1 h at room temperature. The signals were detected by Odyssey infrared imaging system (Li-Cor, Lincoln, NE, USA).

For co-immunoprecipitation analysis, lysates obtained from treated cells were added primary antibody for a 2 h incubation at room temperature with gently rocking, and then incubated with 50% slurry protein A-agarose (cat. no. 11134515001, Roche) overnight at 4 °C. After centrifugation at 12,000×*g* for 10 s, the precipitation was denatured with adding 2× laemmli sample buffer (#1610737EDU, Bio-Rad) and prepared for further immunoblotting analysis.

### Cell viability assay and cytotoxicity LDH release assay

TNBC cells of 5  × 10^3^ per well were cultured in 96-well microplate for 24 h. The cells were randomly divided into several groups and treated with indicated drugs for 24 h. Cell viability was assessed with CCK-8 (CK04, Dojindo Laboratories) according to the manufacturer’s instructions. OD_450_ was measured with a microplate reader (infinite M200, Tecan), to determine the viability of the cells. LDH release was assessed with cytotoxicity LDH assay Kit-WST (CK12, Dojindo Laboratories) after 24 h post-treatment according to the manufacturer’s instructions. OD_490_ was measured to determine the cytotoxicity of the drugs.

### Colony formation assay

Cells were triply seeded at a density about 300 cells per well in six-well plates. After culture for 24 h, cells were treated with drug vehicle (DMSO), BEZ235 (1 μM), and/or ABT263 (1 μM) for 96 h, then the cells were further cultured without drugs for about a week. Once formed, colonies were stained with crystal violet staining solution for obtaining images.

### Drug synergy analysis

Drug synergy analysis was performed via the Chou–Talalay method^22^. Combination index values, which reflect whether there is a synergic effect of two drugs, were calculated using Compusyn software. CI < 1 means synergism, CI = 1 respresents additive effects, and CI > 1 indicates antagonism.

### BAX translocation assay

BAX translocation assay was performed as previously described^30^. Briefly, TNBC cells were co-transfected GFP-BAX and DesRed-mit plasmids for 24 h, and then treated with no drugs, BEZ235 (1 μM), and/or ABT263 (1 μM). Living cell images were obtained using confocal microscope (LSM 510, ZEISS) at 37 °C with 5% CO_2_.

### JC-1 staining assay

JC-1 staining was performed according to the manufacturer’s instructions. After treatment with no drugs, BEZ235 (1 μM), and/or ABT263 (1 μM) for 24 h, MDA-MB-231 cells were stained with mitochondrial membrane potential assay kit with JC-1 (c2006, Beyotime Biotechnology), and measured by FACSCanto II flow cytometer (BD Bioscience).

### Apoptosis analysis

Apoptosis assays were performed as described previously^40^. Cells following indicated treatments were collected and stained with Annexin V, FITC Apoptosis Detection kit (AD10, Dojindo), and measured by FACSCanto II flow cytometer (BD Bioscience).

### RNA analysis

Total RNA was extracted from MDA-MB-231 cells using the RNAiso Plus (Takara). For polymerase chain reaction (PCR) analysis, complementary DNA was synthesized using ReverTra Ace PCR RTMaster Mix with genomic DNA Remover (Toyobo). The specific primer pairs: 5′-TTGTG CTGGTGCCCGTTCCA-3′ (forward) and 5′-AGGCTAGT GGTCACGTTTGGCT-3′ (reverse) were used for detecting *PUMA* while 5′-TCACCCACACTGTGCCCATCTACGA-3′ (forward) and 5′-CAGCGGAACCGCTCATTGCCAAT GG-3′ (reverse) for detecting *Actin*.

### Animal study

Female 5- to 6-week-old nude mice used in this study were housed in a specific pathogen-free environment with micro isolator cages and a 12 h dark–light cycle. This study was performed in accordance with the guidelines of the Guide for the Care and Use of Laboratory Animals (Institute of Laboratory Animal Resources, Commission on Life Sciences, National Research Council). All mice studies were approved by the Institutional Animal Care and Use Committee of South China Normal University (Guangzhou, China). To establish a xenograft model, MDA-MB-231 cells (2 × 10^6^) were suspended in PBS and mixed at a ratio of 1:1 with Matrigel Matrix (356234, CRONING) and then subcutaneously injected into the flanks of nude mice. Once the tumor volumes reached ~200–300 mm^3^, mice were randomized into four groups and treatments began. BEZ235 was dissolved in NMP/PEG300 (1:9) to a concentration of 7 mg/ml and ABT263 was supplied as a stock solution (25 mg/ml in DMSO). BEZ235 (15 mg/kg) or its vehicle was administrated by oral gavage daily^41^ for 4 weeks and ABT263 (25 mg/kg) or its vehicle was injected i.p. three times per week^42^ for 4 weeks. The dose of indicated drugs used for each mouse treatment was calculated according to its body weight. Tumor growth was monitored by electronic calipers every 2 days for 3 weeks, and tumor volumes were calculated according to the formula: *v* = (maximum diameter) × (minimum diameter)^2^/2. Tumor fragments and the organs of heart, lung, liver, spleen, and kidney were collected and snap-frozen after formaldehyde fixation for further analysis.

### Immunohistochemical analysis

Tumor tissues were collected and then fixed in 10% formaldehyde for 24 h. Following dehydration in 75% ethanol solution for 8 h, tissues were embedded in paraffin, and sectioned. The tumor sections were incubated with primary antibodies overnight and secondary antibodies (conjugated Horseradish Peroxidase) for 1 h at room temperature after some steps performed as previously described^43^. Finally, the images were obtained by optical microscope following DAB staining and slightly haematoxylin staining.

### Statistical analysis

All assays were performed a minimum of three times. The data are expressed as the mean ± SEM. Statistical analysis was performed using Student’s paired *t* test, and the differences were considered statistically significant as *P* < 0.05.

## Electronic supplementary material

Supplementary information
