# Supplementary material for: Downregulation of MCL-1 and upregulation of PUMA using mTOR inhibitors enhance antitumor efficacy of BH3 mimetics in triple-negative breast cancer
Source: Cell Death Dis. 2018 Jan 26;9(2):137. doi: 10.1038/s41419-017-0169-2 (PMC5833778; doi:10.1038/s41419-017-0169-2)
Supplement: Supplementary file 1 — Supplementary information [file 41419_2017_169_MOESM1_ESM.pdf]

Supplementary information

Down-regulation of MCL-1 and up-regulation of PUMA using mTOR inhibitors enhance antitumor efficacy of BH3 mimetics in Triple Negative Breast Cancer

Haolong Li [a,b], Lei Liu [a,b,*], Haocai Chang [a], Zhengzhi Zou [a,b] and Da Xing [a,b,*]

[a] MOE Key Laboratory of Laser Life Science & Institute of Laser Life Science, College of Biophotonics, South China Normal University, Guangzhou, 510631, China

[b] Joint Laboratory of Laser Oncology with Cancer Center of Sun Yat-sen University, South China Normal University, Guangzhou, 510631, China

*Correspondence:

Da Xing

Phone: +86-20-85210089; Fax: +86-20-85216052; E-mail: xingda@scnu.edu.cn

Or Lei Liu

Phone: +86-20-8521436; Fax: +86-20-85216052; E-mail: liulei@scnu.edu.cn

Running title: mTOR inhibitors sensitize TNBC to BH3 mimetics

Supplementary materials and methods

Time-lapse confocal fluorescence microscopy for monitoring BAX translocation.

For monitoring BAX translocation, a time-lapse confocal fluorescence microscopy was used as previously described.[1] After co-transfected GFP-BAX and DesRed-mit plasmid for 24 h, MDA-MB-231 cells were treated with BEZ235, ABT263, or their combination. Living cell images were obtained using a time series program in confocal microscope (LSM 510, ZEISS) at 37°C with 5% CO_2.

References

1. Liu L, Xing D, Chen WR. Micro-calpain regulates caspase-dependent and apoptosis inducing factor-mediated caspase-independent apoptotic pathways in cisplatin-induced apoptosis. *International journal of cancer* 2009, **125**(12)**:** 2757-2766.

Supplementary Figures

Figure S1

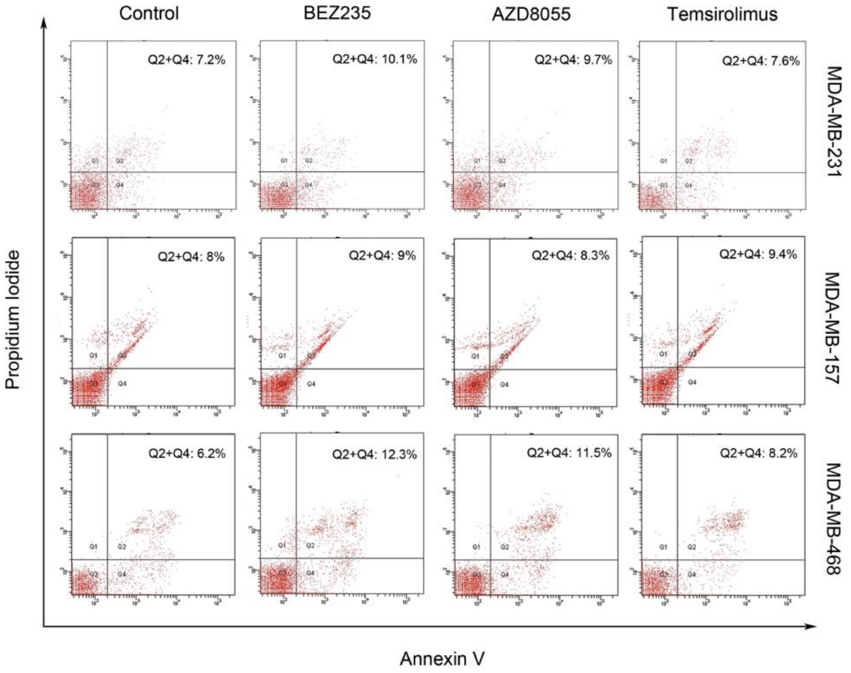

Figure S1 mTOR inhibitors fail to induce obvious apoptosis in different TNBC cells. MDA-MB-231, MDA-MB-157 and MDA-MB-468 cells were treated with indicated mTOR inhibitors (1 μM) for 24 h, and apoptosis analysis was performed by using Annexin V-FITC/PI staining.

Figure S2

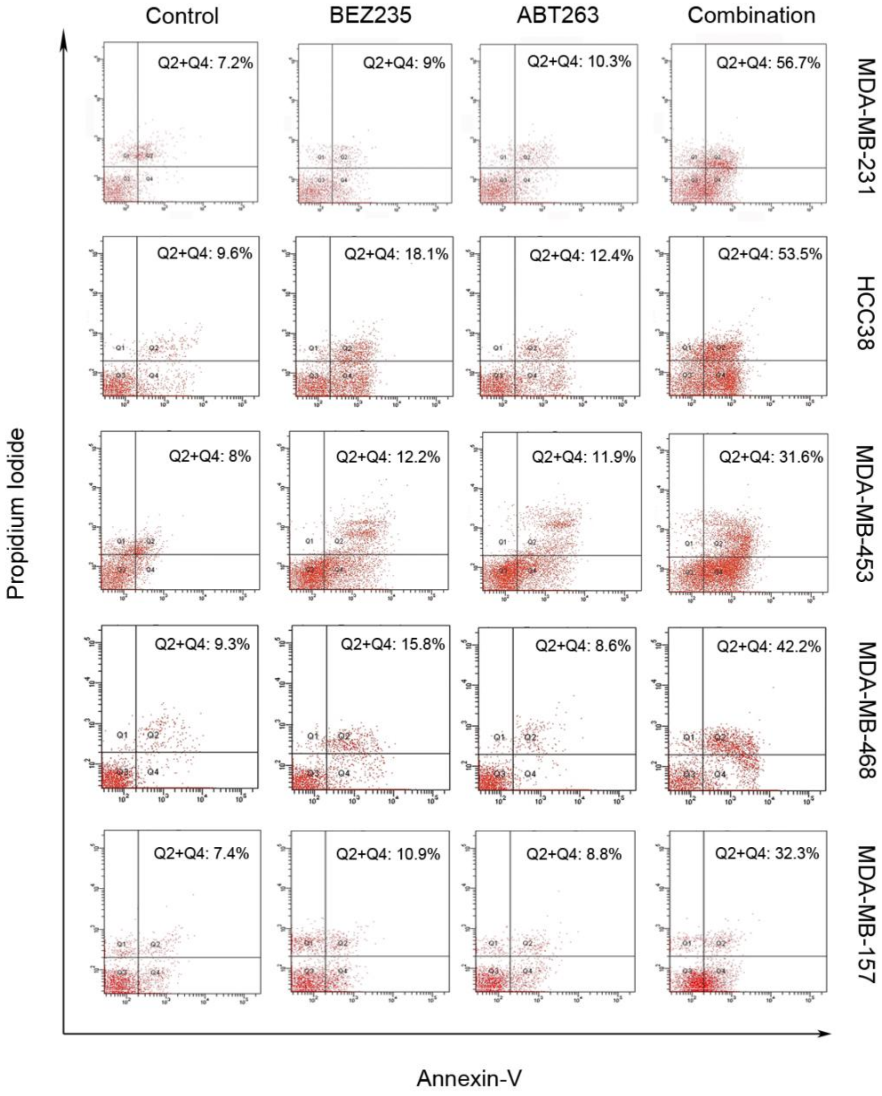

Figure S2 Suppression of MCL-1 sensitizes TNBC cells to ABT-263. Apoptosis analysis of different TNBC cells treated with no drug (control), BEZ235 (1 μM) and/or ABT263 (1 μM) for 24 h.

Figure S3

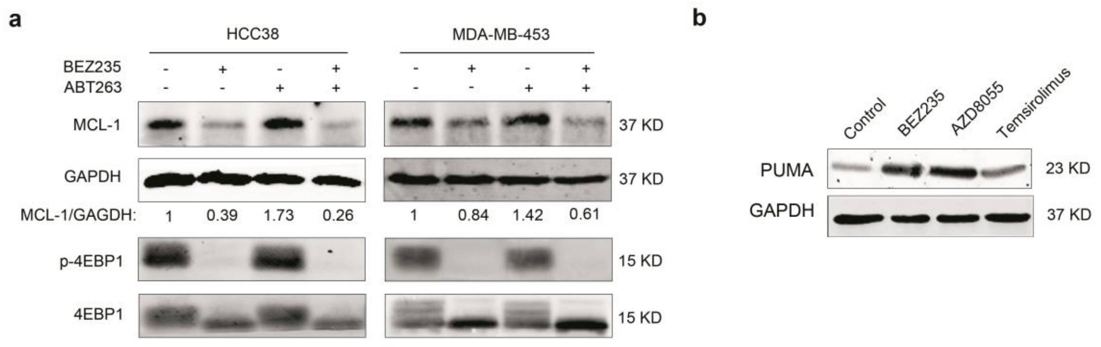

Figure S3 BEZ235 suppresses MCL-1 and up-regulates PUMA. (**a**) Immunoblotting analysis of MCL-1 and p-4EBP1 in HCC38 and MDA-MB-453 cells following treating with no drug (control), BEZ235 (1 μM) and/or ABT263 (1 μM) for 24 h. GAPDH served as loading control. (**b**) Immunoblotting analysis of expression level of PUMA in MDA-MB-231 cells following different mTOR inhibitors (1 μM) treatment.

Figure S4

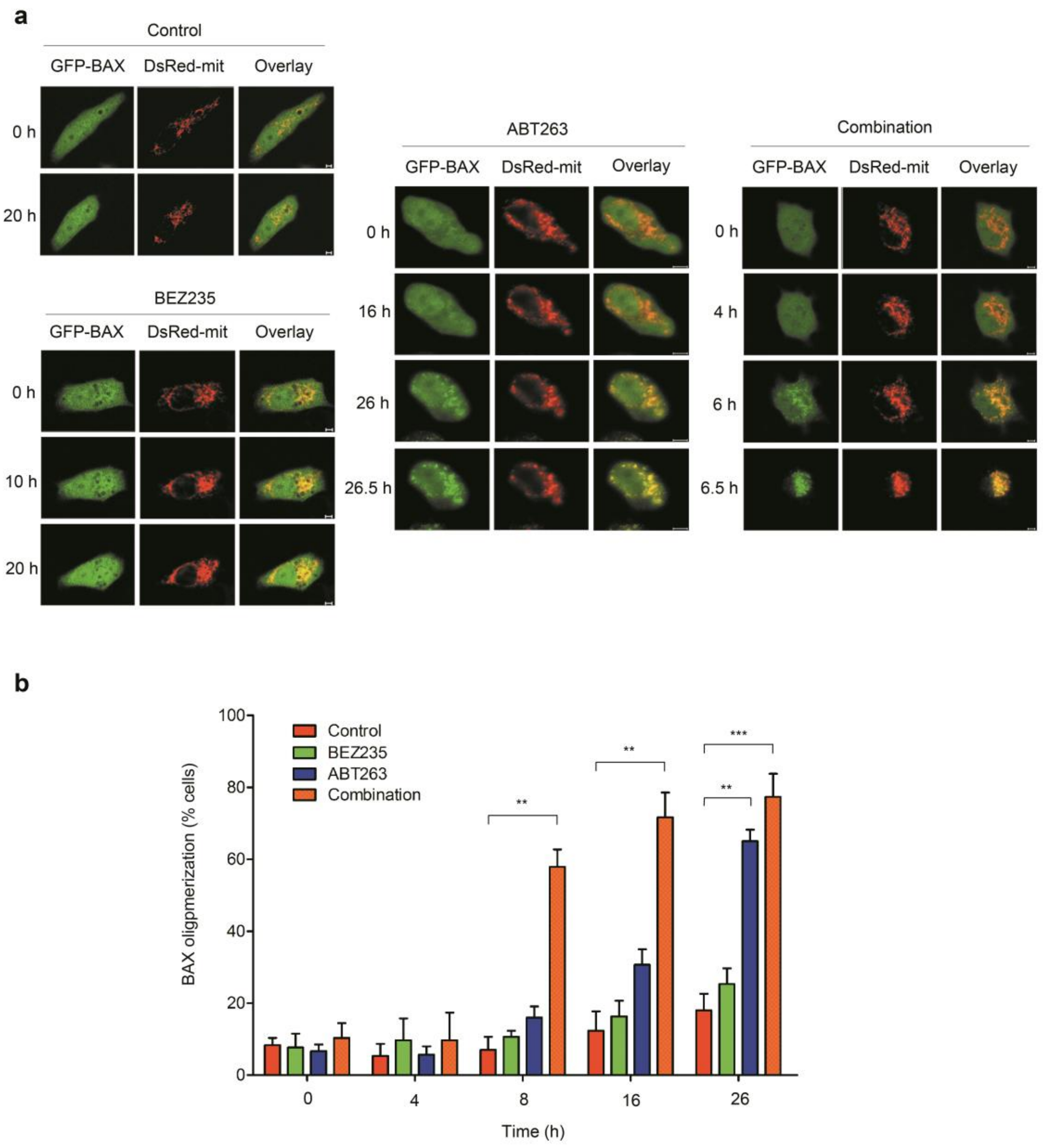

Figure S4 Combination of BEZ235 and ABT263 induces BAX redistribution. (**a**) Real-time detection of BAX translocation under indicated treatment. Typical time-lapse confocal images of MDA-MB-231 cells co-transfected with GFP–BAX and DsRed-mit following different treatments are displayed, bar = 5 μm. (**b**) Quantification of cells showing BAX oligomerization. After different treatments as indicated, the percentage of cells showing BAX oligomerization was assessed by counting the number of cells

exhibiting mitochondrial BAX. $^{**}P < 0.01$ and $^{***}P < 0.001$ *vs.* indicated control group.

Figure S5

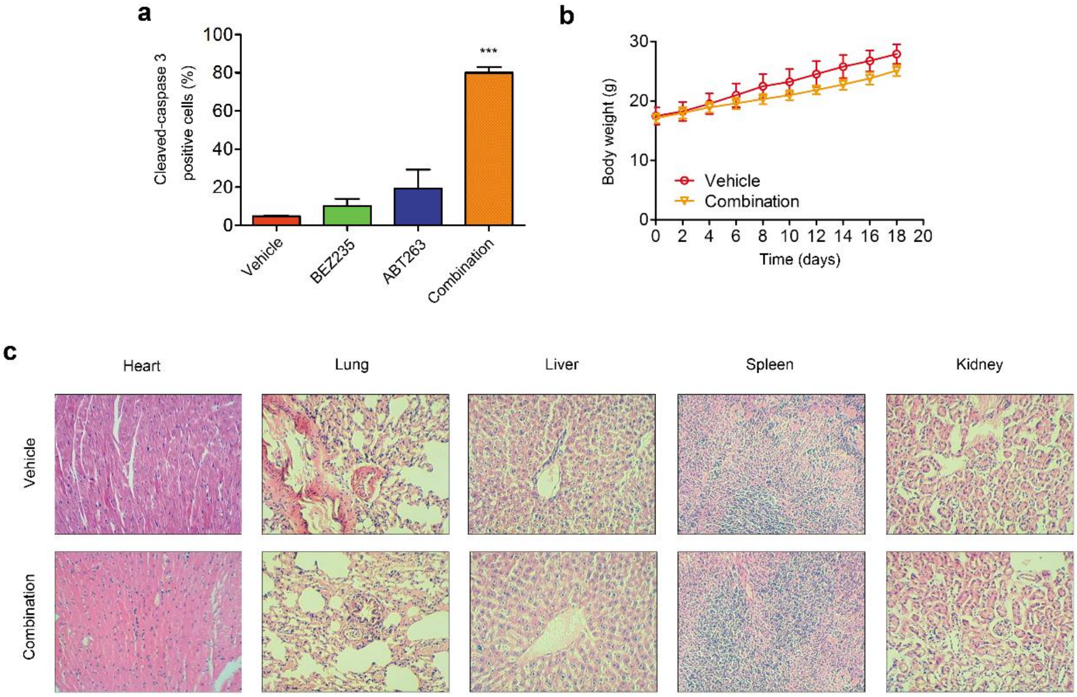

Figure S5 The combination therapy of BEZ235 and ABT263 potently represses tumor growth *in vivo*. (**a**) Quantity analysis of CC3-positive cells shown in Figure 5d. ***$P <$ 0.001 *vs.* control group. (**b**) MDA-MB-231 xenografts were treated with vehicle, BEZ235 (15mg/kg) and/or ABT263 (25mg/kg), and body weights of mice were measured every two days over 3 weeks. Error bar represents SEM ($n \geq 4$). (**c**) H&E staining analysis of different organs from mice following indicated treatments. Representative images are shown (magnification: 20×).
